# Continuous Modeling of T CD4 Lymphocyte Activation and Function

**DOI:** 10.3389/fimmu.2021.743559

**Published:** 2021-11-05

**Authors:** David Martínez-Méndez, Luis Mendoza, Carlos Villarreal, Leonor Huerta

**Affiliations:** ^1^ Instituto de Física, Universidad Nacional Autónoma de México, Mexico City, Mexico; ^2^ Instituto de Investigaciones Biomédicas, Universidad Nacional Autónoma de México, Mexico City, Mexico; ^3^ Centro de Ciencias de la Complejidad, Universidad Nacional Autónoma de México, Mexico City, Mexico

**Keywords:** T CD4 cells, metabolism, T cell receptor, lymphocyte activation, CTLA-4, mTOR, regulatory network, mathematical model

## Abstract

T CD4+ cells are central to the adaptive immune response against pathogens. Their activation is induced by the engagement of the T-cell receptor by antigens, and of co-stimulatory receptors by molecules also expressed on antigen presenting cells. Then, a complex network of intracellular events reinforce, diversify and regulate the initial signals, including dynamic metabolic processes that strongly influence both the activation state and the differentiation to effector cell phenotypes. The regulation of cell metabolism is controlled by the nutrient sensor adenosine monophosphate-activated protein kinase (AMPK), which drives the balance between oxidative phosphorylation (OXPHOS) and glycolysis. Herein, we put forward a 51-node continuous mathematical model that describes the temporal evolution of the early events of activation, integrating a circuit of metabolic regulation into the main routes of signaling. The model simulates the induction of anergy due to defective co-stimulation, the CTLA-4 checkpoint blockade, and the differentiation to effector phenotypes induced by external cytokines. It also describes the adjustment of the OXPHOS-glycolysis equilibrium by the action of AMPK as the effector function of the T cell develops. The development of a transient phase of increased OXPHOS before induction of a sustained glycolytic phase during differentiation to the Th1, Th2 and Th17 phenotypes is shown. In contrast, during Treg differentiation, glycolysis is subsequently reduced as cell metabolism is predominantly polarized towards OXPHOS. These observations are in agreement with experimental data suggesting that OXPHOS produces an ATP reservoir before glycolysis boosts the production of metabolites needed for protein synthesis, cell function, and growth.

## 1 Introduction

The activation of T CD4 lymphocytes is triggered through the proper binding of the T-cell receptor (TCR) to specific antigens presented in the context of the major histocompatibility complex (MHC) on antigen presenting cells (APC), and of co-stimulatory molecules like CD28 with ligands such as CD80 and CD86 (jointly denoted as CD80/86), also displayed on the APC membrane. Activation involves the coordinated activity of a plethora of intra- and extra-cellular biochemical mediators forming a network that reinforces, amplifies, diversifies, and regulates the initial antigenic and co-stimulatory signals (1–9). It is known that TCR activation is a progressive process, since MHC–peptide molecules serially engage several TCRs, amplifying the magnitude of intracellular signals that eventually cross a certain activation threshold (10). Furthermore, a minimal interaction half-time between the TCR and MHC is required for productive TCR signaling (10–13). Under optimal stimulation, activation ultimately leads to cell proliferation and differentiation into particular effector cell phenotypes, which are active against diverse antigens. In contrast, binding of antigen to the TCR in the absence of CD28 ligation conduces to a state of anergy. In such a state, T cells are unable to produce interleukin 2 (IL-2) or proliferate on subsequent stimulations (14–16).

Activation of T CD4 cells by antigen and co-stimulatory molecules triggers inhibitory pathways that regulate the whole process, among which the cytotoxic T-lymphocyte antigen 4 (CTLA-4), expressed on the surface of activated T cells, has been the most extensively investigated (17, 18). CTLA-4 is partially homologous to CD28 and binds to the same ligands (CD80/CD86) on the APC, although with a much higher affinity than the latter. Thus, upregulation of this molecule on activated cells results in the competition between CD28 and CTLA-4 for binding to CD80/CD86 (19, 20). The displacement of CD28 by CTLA-4 induces of a state of cellular arrest known as checkpoint blockade (21–24).

**Figure 1:**
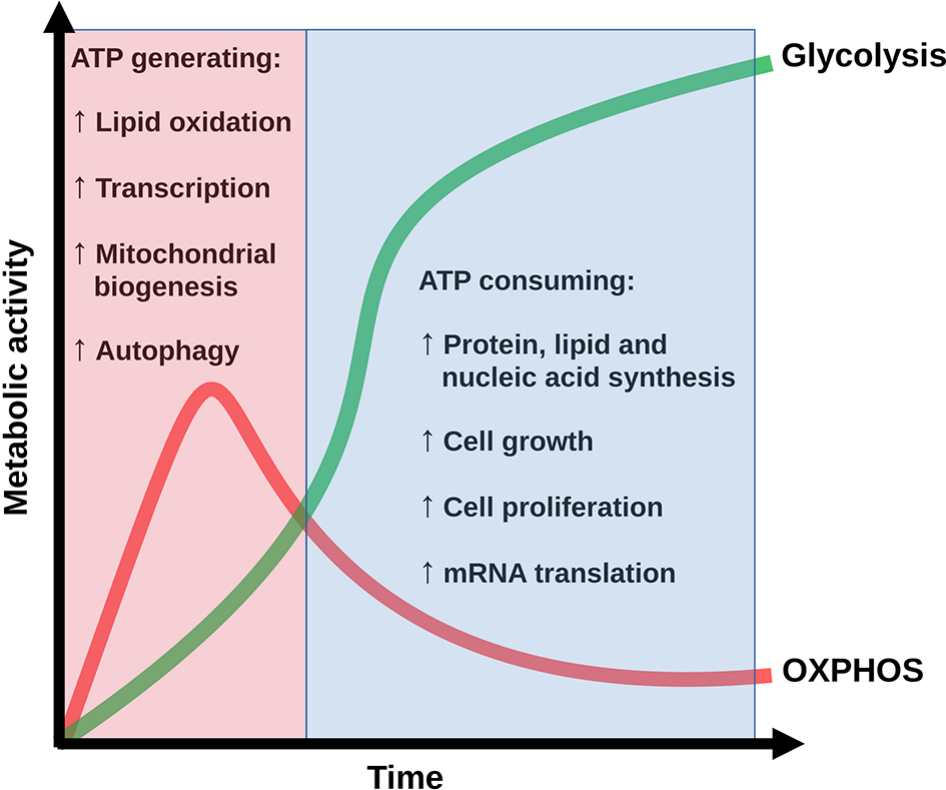
Conceptualization of the time evolution of the OXPHOS-glycolysis shift along the bioenergetic and biosynthetic profile of T CD4 cells under antigenic stimulation. Adapted from (25).

The activation process intrinsically involves the function of metabolic mediators whose activity is necessary to fulfill the bioenergetic and biosynthetic demands of increased cell proliferation and function (9, 25, 26). Metabolism of resting naïve cells depends on the tricarboxyic acid (TCA) cycle linked to oxidative phosphorylation (OXPHOS), a highly efficient but slow route for ATP generation. Upon activation, T cells rapidly shift to a predominant glycolytic metabolism, a less efficient process of ATP generation which, however, produces essential molecular intermediates for the generation of metabolites required for growth and proliferation [reviewed in (27)]. In spite of abundant information on the metabolic shift toward glycolysis, evidence suggests that OXPHOS is also induced in early states of activation, since AMPK is activated by signals from the TCR, CD28 and Ca^2+^ (2, 25, 28–30) (**Figure 1**). A general theoretical model is necessary to integrate the pathways of signaling from the TCR and costimulatory molecules with those from metabolic controllers, in order to understand how the balance between glycolysis and OXPHOS is established and how it is adjusted as effector functions arise.

A number of mathematical models have been proposed to analyze the intricate organization underlying the general mechanisms involved in T cell response (31–40), as well as the role of central actors participating in the regulation of the immune response (41–43). In this context, we previously addressed the early intracellular events in T cell activation and subsequent cell differentiation by analyzing the dynamics of a 46-node hybrid Boolean model. The model was based on a network composed by a central module simulating the activation induced by priming of TCR and CD28, another module describing the CTLA-4-mediated regulation of activation, and four modules corresponding to the events inducing differentiation to the Th1, Th2, Th17, and Treg phenotypes (40).

With the purpose of modeling the mutual regulatory mechanisms of T CD4 lymphocyte activation and metabolism, in the present work we put forth a sub-network simulating the main processes of cellular metabolic control by AMPK; this module has been subsequently incorporated into the formerly described network to achieve an integrated scheme of the immune and metabolic processes driving the early events of T cell activation. In addition, two nodes have been introduced to represent the time-dependent priming of the TCR by MHC-antigen, as well as the competitive engagement of CD28 or CTLA-4 to the CD80/CD86 complex. As a preliminary step, the resulting 51-node network was characterized in terms of a set of discrete Boolean rules determining the fundamental interactive topology of the system. However, a more realistic description should take into account that the expression levels, concentrations, and parameters of the system may display any value within a continuous range limited only by functionality constraints. Thus, we performed the translation of the discrete interactive Boolean rules to the continuous domain through an algorithmic approach based on fuzzy logic. Fuzzy logic is a theory aimed to provide formal foundation to approximate reasoning (44, 45). Applied to biological systems, fuzzy propositions describe cases in which a cell displays intermediate levels of expression/activity of elements, so that they do not necessarily belong to a specific phenotype (46). The fuzzy logic rules were introduced as inputs into a system of ordinary differential equations to describe the overall network dynamics. The continuous analysis allows the introduction of variable degrees of activating stimulus and the description of gradual changes of the output elements reflecting activation. It also allows to assess the influence of different time-scales of activity of key components of the signaling network.

## 2 Methods

### 2.1 Inference and Integration of a Network Module of Metabolism Control

A concise network of the main components controlling T cell metabolism was constructed based on experimental information. A central actor in the lymphocyte metabolic activity is the AMPK complex, which is capable of sensing the intracellular AMP/ATP ratio, which represents the T cell energy pool availability, and of regulating the main metabolic pathways leading to the production of energy reserves (OXPHOS) or to the rapid generation of metabolites and structural proteins (glycolysis) (47–52). It has been demonstrated that two main effects related to metabolism take place upon TCR stimulation and CD28 co-stimulation (53). First, an increase in the basal activity of oxidative phosphorylation (OXPHOS) arises promoted by the action of the nutrient sensor AMPK, which is activated directly by the PI3k-AKT axis and calcium release (54). Afterwards, mTORC1 is activated with the consequent inhibition of AMPK and the activation of glycolysis (55).

In the network, AMPK is activated in several ways: signaling from the TCR and CD28 *via* the PI3k-AKT axis, calcium release, a high AMP/ATP ratio, the activation of the serine–threonine liver kinase B1 (LKB1) and the Foxp3 transcription factor (7, 47, 48, 56). Although there are several signaling intermediates proposed in these pathways, they were mathematically implied due to the lineal nature of the signaling trajectory in order to obtain a set of simplified logical propositions.

The balance between OXPHOS and glycolysis depends primordially on a negative feedback loop between AMPK and mTORC1, which plays the role of a metabolic polarization switch (50, 52, 57, 58). In the network presented here, TCR and CD28 activation, an elevated AMP/ATP ratio, LKB1 and Foxp3 activate AMPK, thus inhibiting the activity of mTORC1, finally leading to OXPHOS and inhibition of glycolysis.

**Figure 2:**
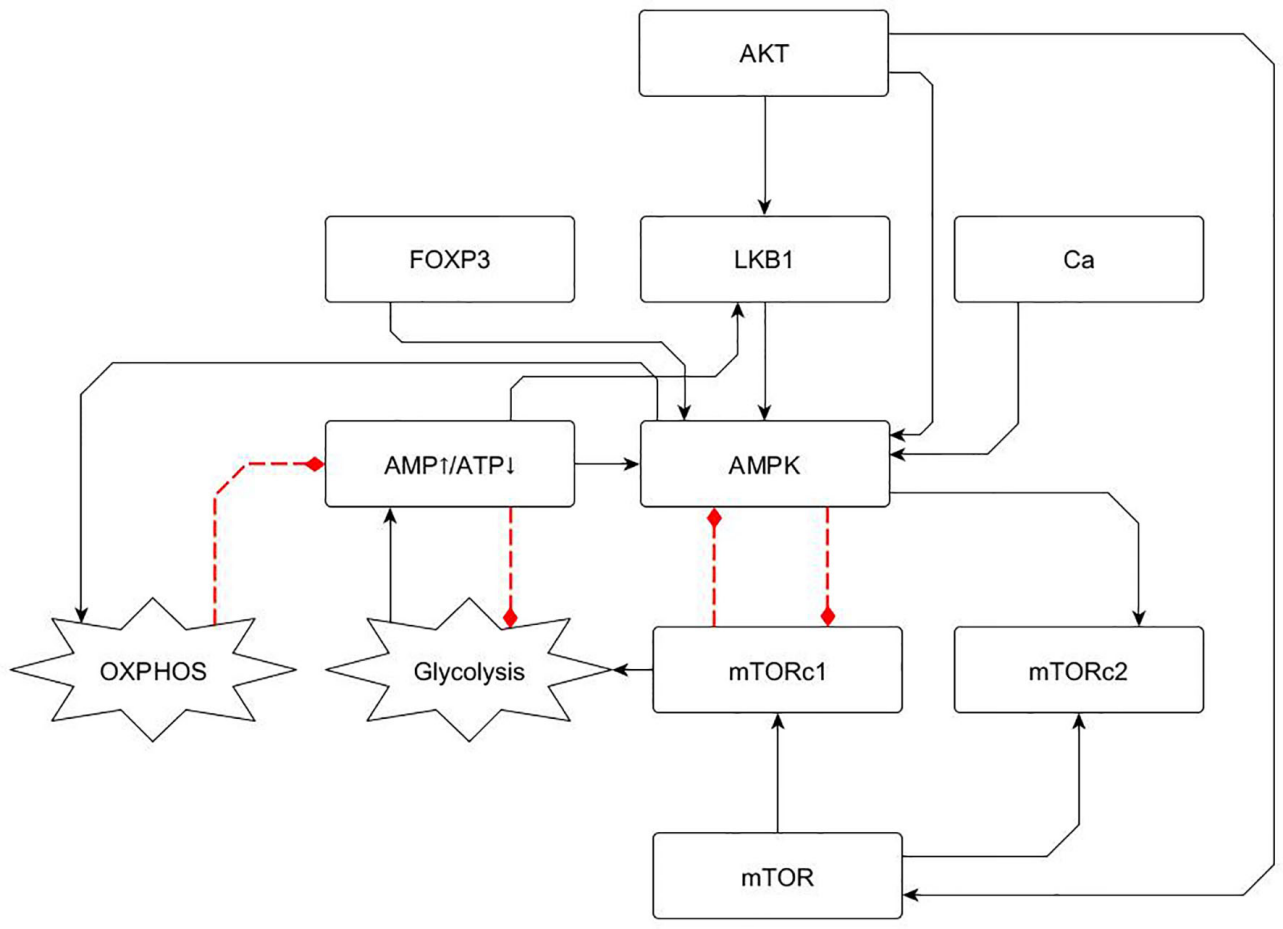
Metabolism module including basic elements involved in glycolysis and OXPHOS regulation, as described in section 2.1. AMPK is a central energy sensor of the AMP/ATP ratio and displays a negative feedback loop with MTORC1. This loop defines a switch driving either OXPHOS or glycolytic activity. Continuous and dotted lines represent activator and inhibitory pathways, respectively.

**Figure 3:**
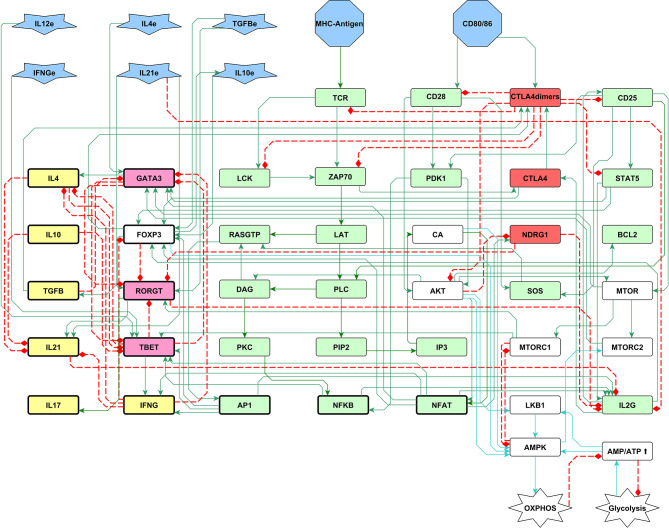
A 51-node network of the early biochemical interactions induced by TCR activation, co-stimulation, and phenotype-inducing cytokines. Inputs of the network are the MHC-antigen, the co-stimulatory molecules CD80/86, and external cytokines (blue polygons). The set of interacting rules defining the dynamical system for Boolean modeling is shown in **Supplementary Material 1**. Boolean rules were translated to continuous functions as shown in Material and Methods, and the complete set of ordinary differential equations is provided in **Supplementary Material 2**. Green and red rectangles represent stimulatory and inhibitory nodes of the activation core, respectively. Pink and yellow rectangles represent phenotype-inducing transcription factors and cytokines produced endogenously, respectively. White rectangles represent the metabolism module including AMPK as a central regulatory element, as shown in figure 2. Construction of the network can be consulted in (40). LAT: LAT-Gads-SLP-76 complex; IL2G: IL-2 gene; L4e, IFNγe, IL10e, TGFβe and IL21e: exogenous cytokines.

Conversely, under a low AMP/ATP ratio, AMPK is inhibited, which in turns activates mTORC1 function and additional inhibition of AMPK, leading to glycolysis (**Figure 2**). The metabolic module was then incorporated into the previously reported Boolean network of T CD4 cell activation through links associated to AMPK and mTOR (**Figure 3**). The network previously constructed encompasses modules corresponding to an activation core derived from TCR and CD28 signaling, a feedback loop for IL-2 production though its high-affinity receptor (CD25), the role of the anergy factor NDRG1 (controlled by AKT), activation of the checkpoint by CTLA-4, and the induction of the effector phenotypes by external cytokines (59, 60). The construction of the network can be consulted in (40).

### 2.2 Boolean Approach

The interactions involved in the metabolism module were formalized as Boolean propositions as shown in **Table 1**. After incorporation into the general T-cell activation network proposed in (40), an exhaustive analysis was performed to analyze its general behavior and congruence with our previously published results (**Supplementary Material 3**). The robustness of the integrated model was also verified by introducing random noise in the initial states and measuring the distance between transition states and attractors. Robustness was also tested by either inducing perturbations in the network structure by random bit flipping of the Boolean functions or by random permutation of the output values (data not shown). The set of functions that defines the whole activation network are shown in **Supplementary Material 1** and the model structure can be consulted in Wolfram Notebook format in https://github.com/DrDavidMM/TCD4cell-activation-model-supplementary-material-.git.

**Table 1:** Boolean rules for the metabolism module. Here, ∨→ or, ∧→ and, while ¬→ not.

VARIABLE	TRANSITION RULE
*MTOR* (*t* + 1) =	*CD*25 (*t*) ∨ AKT (*t*)
*MTORC*1 (*t* + 1) =	*MTOR *(*t*) ∧ ¬*AMPK *(*t*)
*MTORC*2 (*t* + 1) =	[*MTOR *(*t*) ∧ *AMPK *(*t*)] ∨[*MTOR *(*t*) ∧ *IL*4*e* (*t*)]
*LKB*1 (*t* +1) =	*AKT *(*t*) ∧ *AMP*/*ATP *(*t*)
*AMPK* (*t* + 1) =	[*LKB*1(*t*) ∧ ¬*MTOR*C1 (*t*)] ∨[*CA *(*t*) ∧ *AMP*/*ATP *(*t*) ∧ ¬*MTORC*1 (*t*)]
	∨[*AKT *(*t*) ∧ *AMP*/*ATP*(*t*) ∧ ¬*MTORC*1(*t*)] ∨*FOXP*3 (*t*)
Gycolysis(*t* + 1) =	*MTORC*1 (*t*) ∧ ¬ *AMP*/*ATP *(*t*)
*OXPHOS*(*t* + 1) =	*AMPK* (*t*)
*AMP*/*ATP*(*t* + 1) =	*Glycolysis *(*t*) ∧ ¬*OXPHOS *(*t*)

### 2.3 Continuous Fuzzy Logic Approach

Fuzzy logic is characterized by a graded approach, so that the degree to which an object exhibits a given property is specified by a membership (or characteristic) function with truth values ranging from total falsity, *µ*[w*_k_*] = 0, to totally true, *µ*[w*_k_*] = 1. Here, w*_k_* denotes a fuzzy logic proposition describing the interactions of node *k* with the rest of network nodes. By assuming that the state of the regulatory network at time *t* is described by the set {q1(*t*),…q*_n_* (t)}, *µ*[w*_k_*], may be represented by a sigmoid function with continuous variation in the interval [0,1]:


(1) $$\mu [w_{k}]=\frac{1}{1+e^{-\beta (w_{k}(q_{1},\ldots ,q_{n})-w_{thr})}},$$

where w*_thr_* is an activation threshold, hereby considered as w*_thr_* = 1/2, and β is a saturation rate (46, 61).

The Boolean interaction rules *W_k_* [*q*_1_(*t*), *q*_2_(*t*), … *q_n_* (*t*)] were translated into fuzzy logic expressions


$$W_{k}[q_{1}(t),q_{2}(t),\ldots \;q_{n}(t)]\to w_{k}[q_{1}(t),q_{2}(t),\ldots \;q_{n}(t)].$$

This procedure can be straightforwardly implemented by replacing the Boolean logic connectors ‘and’, ‘or’ and ‘not’ by their fuzzy counterparts according to the following scheme:

**Table:** 

Boolean	Fuzzy Logic
*q* and *p*	*q*·*p*
*q* or *p*	*q* + *p* – *q*·*p*
not *p*	1 – p

An example of the translation from Boolean to a fuzzy framework is:


W[p,q,r]=(q or p) and (not r)→w[p,q,r]=(q+p−q·p)·(1−r),

Within the continuous scheme, the dynamical behavior is determined by a set of ordinary differential equations describing the temporal change of the activity level of the network components. For the *k* - *th* node, this is written as


(2) $$\frac{dq_{k}}{dt}=\mu [w_{k}(q_{1},\ldots ,q_{n})]-d_{k}q_{k},$$

Where d*_k_* is the decay rate of node *k*. In this work, we assume that and that the default value of d*_k_* = 1, unless otherwise stated. It is important to notice that in absence of an input (w*_k_* = 0), the activity of node *k* decays exponentially, that is, $q_{k}∼e^{-d_{k}t}$ ; therefore, the parameter τ*_k_* = 1/*d_k_* represents a characteristic expression time, so that *d_k_* >1 (*d_k_* <1) gives rise to a relatively rapid (slow) decay of the activity of the element *k* of the network. This kind of analysis allows to assess the influence of different time-scales of activity of the elements comprising the regulatory network.

In the continuous scheme, the equilibrium states (attractors) of the system are defined by the condition *dq_k_*/*dt* = 0. This condition implies that, for a specific set of initial values {*q*_1_(0),…,*q_n_* (0)}, the system dynamically evolves until reaching steady-state values given by:


(3) $$q_{k}^{st}=\frac{1}{d_{k}}\mu [w_{k}(q_{1}^{st},\ldots ,q_{n}^{st})].$$

This latter expression shows that the resulting set of asymptotic states, $\left\{q_{1}^{st},\ldots q_{n}^{st}\right\}$ , is determined, besides the initial values *q_k_* (0), by the actual values of the decay rates *d_k_*. A consequence of the former results is that the emergent behavior of the system may conduce to alternative dynamic patterns depending on the specific values of *i* ) the set of initial concentrations, dosages, or expression levels, {*q_k_* (0)}, and *ii*) differences in either stimulation times, τ*_stim_*, or characteristic expression times of the network components given by their decay rates, τ*_k_* ~ 1/*d_k_* (46, 61, 62).

### 2.4 TCR-Antigen, CD28-CD80/86, and CD28-CTLA-4 Interactions

The extent and time span of stimulation of T CD4 cells due to MHC-antigen presentation to TCR and of CD80/86 binding to CD28, was broadly modeled by introducing two input nodes whose priming activity only lasts for a limited lapse of time τ*_stim_*. This was described by means of functions with a step-like behavior associated to an initial and constant avidity strength, A*_MHC/A_* and A*_CD_*_8086_, suffering an abrupt decay at time *t* = τ*_stim_* by a factor D*_MHC/A_* and D*_CD_*_8086_, respectively. The time variations of avidity can be related to changes in the number of TCR-MHC-peptide complexes, the presence of adhesion molecules, TCR internalization or degradation after initial engagement, etc. (10, 62–67). Now, by introducing the step-function *H*(*t* − τ*_stim_*) defined by


$$H(t-\tau_{stim})=0,\;if\;t-\tau_{stim}<01,\;if\;t-\tau_{stim}>0$$

then the avidity variations are written as follows:


(4) $$A_{MHC/A}(t)=A_{MHC/A}-D_{MHC/A}H(t-\tau_{MHC/A})$$

(5) $$A_{CD8086}(t)=A_{CD8086}-D_{CD8086}H(t-\tau_{CD8086}),$$

where *D_MHC/A_* and *D_CD_*_8086_ represent the magnitude of detachment reduction of TCR and CD28 from their ligands for times longer than τ*_MHC/A_* and τ*_CD_*_8086_, respectively.

This approach does not only allow to analyze the behavior of the cell as a function of the extent of stimulation, but also the description of the competitive action between CD28 and CTLA-4 for binding to CD80/86. This is performed through the downstream interactions of CD28 with CTLA-4 (see **Figure 3**). Upon activation, CTLA-4 may down-regulate the engagement of CD28 with CD80/86. However, its inhibitory capacity depends on its decay rate, *d_CTLA_*_4_, which should be relatively small (<1), in order to have an expression time long enough to overwhelm the influence of factors that promote the transcription factors activity. In the model, longer interaction times of the antigen with the TCR and CD28 allows a sustained activation state before being arrested due to the activity of CTLA-4. This process is simulated by introducing diverse values for the decay rate of CTLA-4, *d_CTLA_*_4_, above and below the default value *d_CTLA_*_4_=1, combined with different temporal duration of antigen attachment, τ*_stim_* (**Figure 5**).

### 2.5 Numerical Methods

The set of differential equations that defines the dynamical system is shown in the **Supplementary Material 2**. The model equations are presented in Wolfram Notebook format in https://github.com/DrDavidMM/TCD4cell-activation-model-supplementary-material-.git.

For the computation of the differential equations system, Wolfram Mathematica 12.2.0.0 and open-source R studio have been used with the packages BoolNet, deSolve and ggplot2. For visual display of the interaction network, we use yEd graph editor 3.20.1 from yWorks. A link to the wolfram cloud public code has been added in the readme file in the GitHub repository.

## 3 Results

A module describing the main processes of cellular metabolic control by AMPK was constructed as described in **Section 2.1** and **Figure 2**, and incorporated into a general T-cell activation network previously described in (40). In addition, two input nodes were introduced to represent the MHC-antigen and CD80/CD86 complexes, the presence of which activates the TCR and either CD28 or CTLA-4, respectively. The whole 51-node network was then reformulated as a set of logical rules and introduced in a system of ordinary differential equations to describe the activation dynamics (**Figure 3**).

An exhaustive analysis of the continuous model was performed to determine the congruence with our previously published results using the hybrid Boolean model **(see section 2.2).** The analysis showed that the attractors obtained in the hybrid Boolean approach can be recovered by the continuous model under certain restrictive conditions, equivalent to assume that the network variables may acquire only values corresponding to null or full expression (0 or 1).

The continuous model allows the introduction of variable levels of stimulating conditions (for example, duration of antigenic priming) and characteristic expression times (inverse decay rates) of the elements constituting the regulatory network. Likewise, it outlines gradual changes in the output elements (like AP-1, NFAT and NFκB transcription factors, and type of metabolism).

### 3.1 Initial Stimulation Conditions: From Naive to Antigen-Primed T Cells

We have assumed that T-cells initially are in a naive state, that is, TCR and CD28 are expressed and not activated. These conditions were implemented by considering that at time *t* = 0, TCR = 0, and CD28 = 0. MHC-antigen and CD80/86 act as inputs activating the TCR and CD28, respectively (although with the course of time CD28 may be displaced by CTLA-4). Optimal stimulation by MHC-antigen and CD80/86 was represented as A*_MHC_*_/_ *_A_* = 1, and A*_CD_*_8086_ =1. Similarly, sub-optimal stimulation was represented by introducing values smaller than unity for either A*_MHC_*_/_ *_A_* or A*_CD_*_8086_ (or both). As shown below, the level of stimulation defined by these parameters can produce activation or anergy. Since the metabolic profile of naive T-cells is characterized by a basal level of OXPHOS, modeling was performed assuming an initial value of OXPHOS = 0.2 and a high AMP/ATP ratio = 1, consistent with a significant activity of the nutrient sensor AMPK = 1.

### 3.2 The AMPK-mTOR Axis Promotes Metabolic Polarization During T CD4 Activation

**Figure 4:**
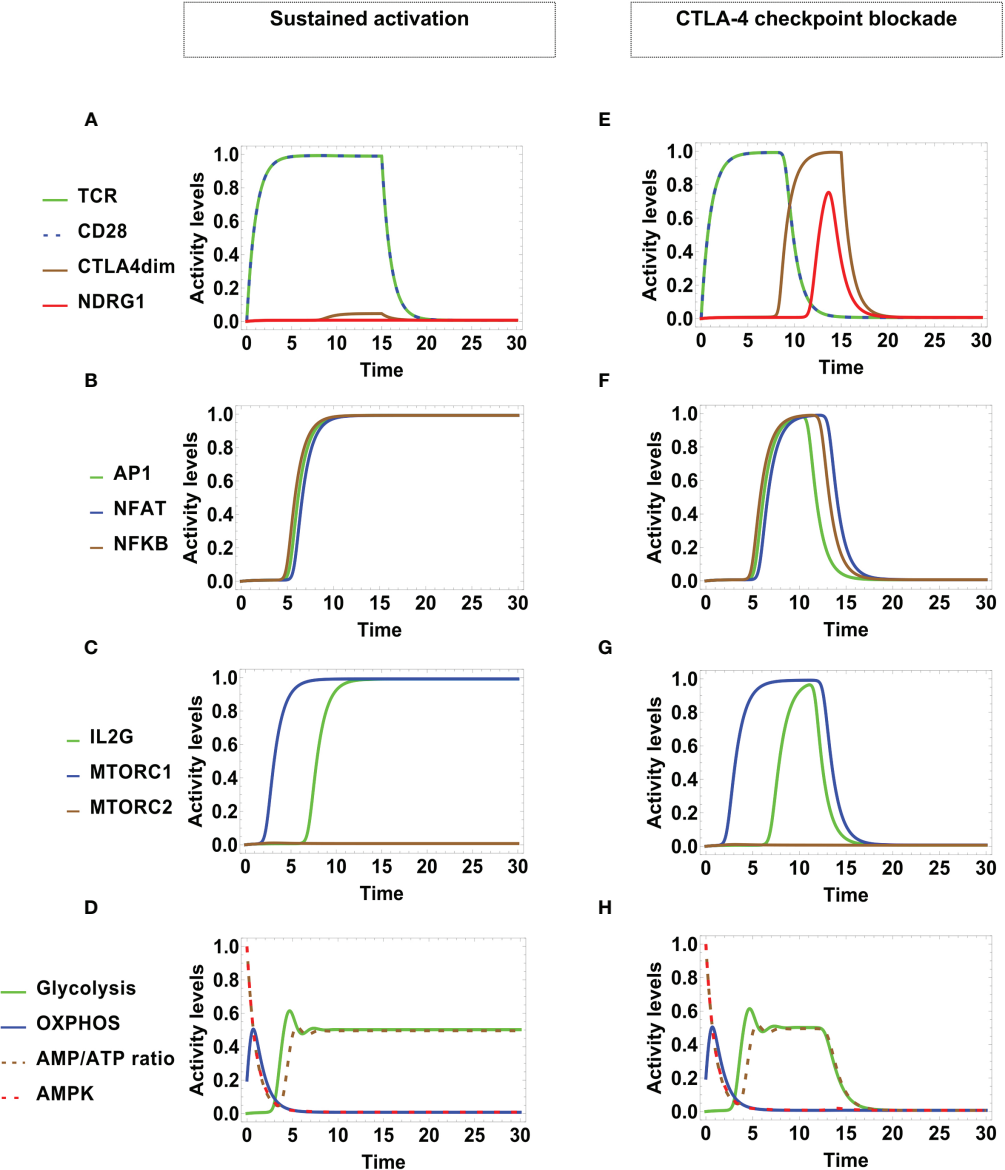
Dynamics of transcription factors and metabolism under optimal engagement conditions of TCR with the MHC-antigen complex, and CD28 with CD80/86, during a stimulation time τ*_MHC_*_/_ *_A_* = τ*_CD_*_8086_ = 15 units. Left panel: Sustained T-cell activation associates to low-level activity of CTLA-4 (with high decay rated *d_CTLA_*_4_ = 5). **(A)** TCR and CD28 are fully activated as far as antigenic stimulation persists, leading to **(B)** sustained expression of the transcription factors AP-1, NFAT, and NFκB, and **(C)** sustained expression of mTORC1 and IL-2. **(D)** Metabolic profile: After initial activation, the AMP/ATP ratio decreases, leading to a reduction of the activity of the nutrient sensor AMPK and a temporary increment of OXPHOS. With the course of time, OXPHOS decays and glycolytic activity increases up to a steady level, in parallel with the AMP/ATP ratio. Right panel: Checkpoint blockade associated to high-level activity of CTLA-4 (with low decay rate *d_CTLA_*_4_ = 0.5). **(E)** Initially, TCR and CD28 are fully activated. With the course of time, the expression level of dimerized CTLA-4 increases, displacing CD28 from co-stimulatory molecules, with the concomitant induction of anergy. This is manifested as **(F)** transitory expression and down-regulation of the activation transcription factors AP-1, NFAT, NFκB, and **(G)** transitory expression and down-regulation of mTORC1 and IL-2. **(H)** Metabolic profile: At the beginning, the metabolic activity shows an identical pattern as in the case of sustained activation; eventually, the inhibitory action of CTLA-4 induces the decay of glycolysis.

Under optimal stimulating conditions, the network dynamics conduces to alternative states of sustained activation or CTLA-4-mediated arrest, each differing in their metabolic profile (**Figure 4**). The presence of MHC-antigen and CD80/86 (at time *t* = 0) induces the activity of TCR and CD28 (**Figure 4A**). These interactions were maintained at a maximal level at stimulation times τ*_stim_* = τ*_MHC_*_/_ *_A_* = τ*_CD_*_8086_ = 15 units, until disengagement was induced at longer times *t* > τ*_stim_*. During the stimulation time, dimerized CTLA-4 is transiently expressed at a low level, which however, is not sufficient to compete with CD28 for binding to CD80/86. Under these conditions, activation leads to the expression of the AP1, NFAT, and NFκB transcription factors at later times (**Figure 4B**) and, accordingly, transcription of the IL-2 gene (IL2G) and MTORC1 are also fully expressed (**Figure 4C**).

The predicted dynamic behavior of the activation process is coherent with trends inferred from the network interactive relationships depicted in **Figures 2** and **3**. As indicated above, we assumed an initial naive state characterized by a low basal level of OXPHOS, a large AMP/ATP ratio, and activity of AMPK (**Figure 4D**). Upon T cell stimulation by TCR and co-stimulatory molecules, AMPK is further activated by Ca^2+^, LKB1 and AKT, boosting an early increase of OXPHOS. After reaching a peak of activity, OXPHOS is undermined due to the decrease of the AMP concentration and the afterward contribution of mTORC1, which impairs the repressive activity of AMPK and leads metabolism polarization towards glycolysis. Glycolysis contributes to the synthesis of cell-growth metabolites and maintenance of the ATP pool. This predicted behavior is congruent with experimental data showing that AMPK is activated early after T cell stimulation, which indicates that the engagement of mitochondrial metabolism is important for exiting quiescence (68), whereas the expression of enzymes pertaining to the glycolytic pathway is dispensable at earlier times (69). Thus, the model is in agreement with the proposal that AMPK activation ensures sufficient ATP availability to progress through full activation (53, 70). It can be observed that, even if TCR and CD28 were stimulated during a limited time span, the production of the AP-1, NFAT and NFκB transcription factors is held longer.

### 3.3 CTLA-4 Checkpoint Blockade

After TCR and CD28 stimulation, in due course the expression level of dimerized CTLA-4 increases, displacing CD28 from co-stimulatory molecules and leading to a state of activation arrest, or checkpoint. Since arrested cells have decreased levels of protein synthesis and expansion, the action of CTLA-4 may also have implications on the regulation of metabolism (71). To model this process, we assumed initial optimal engagement of TCR with the MHC-antigen complex, and CD28 with CD80/86, during an antigen presentation time τ_*MHC*/*A*_ = τ*_CD_*_8086_ = 15 units, combined with a high-level activity of CTLA-4, induced by a low decay rate *d_CTLA_*_4_ = 0.5.

Similar to the previous case, TCR and CD28 are fully activated due antigenic stimulation (**Figure 4E**). However, with the course of time the inhibitory action of CTLA-4 and the anergy factor NDRG1 are expresses, coinciding with the diminution of the levels of activity of TCR and CD28. This leads to an only transitory expression of the AP-1, NFAT, NFκB transcription factors (**Figure 4F**), the IL-2 gene, and mTORC1 activity (**Figure 4G** **)**. The initial metabolic activity shows a pattern similar to that displayed in sustained activation. Nevertheless, the regulatory action of CTLA-4 eventually induces the decay of glycolysis (**Figure 4H**).

### 3.4 Influence of Stimulation Time and CTLA-4 Activity on Sustained or Regulated Activation

The model predicts that cell activation depends on whether the extent of the stimulation time of TCR and CD28, τ*_stim_*, is long enough to overcome the regulatory activity of CTLA-4, which persists during a characteristic time, defined by τ*_CTLA_*_4_ = 1/*d_CTLA_*_4_. The functionality of CTLA-4 resides in its continuous turnover, cellular location, and membrane delivery (18). In combination with activation-promoting factors, in the present model the decay rate of functional CTLA-4 is able to encompass these processes. In the simulation shown in **Figure 4**, sustained activation ensued by assuming that τ*_MHC_*_/_ *_A_* = τ*_CD_*_8086_ = 15, and *d_CTLA_*_4_ = 5, whereas regulated activation was associated to τ*_MHC_*_/_ *_A_* = *τ_CD_*_8086_ = 15, and *d_CTLA_*_4_ = 0.5.

**Figure 5:**
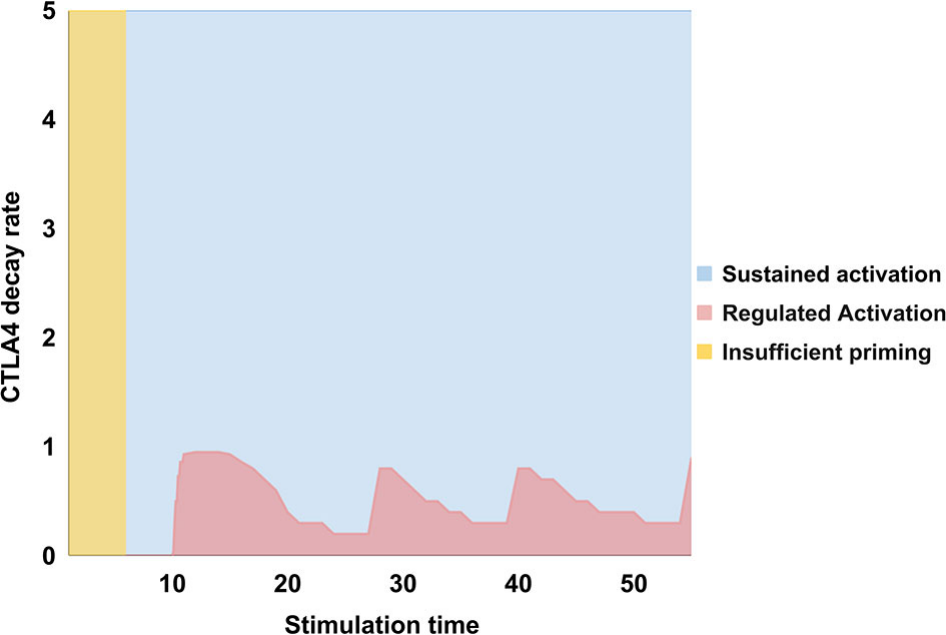
Values of the decay rate of CTLA-4, *d_CTLA_*_4_, as a function of the stimulation time, τ*_stim_* = *t_CD_*_8086_ = *t_MHC/A_*, associated to states of no activation (yellow), sustained activation (blue), or regulated activation (pink). For τ*_stim_* < 7 units no activation arises; sustained activation ensues for 7 ≤ τ*_stim_* ≤ 10, and regulated activation for τ*_stim_* > 10. The first stage implies that a minimum stimulation time is necessary to boost activation; the second one, that CTLA-4 requires a minimal time of activation to be induced and perform inhibition; the third stage reveals that regulated activation only arises at later stimulation times and when the CTLA-4 decay rate is low, that is, *d_CTLA_*_4_ < 1. Therefore, CTLA-4 should be induced for a time long enough to overcome a threshold level and its activity should be maintained to perform inhibition. The oscillatory behavior of the regulation threshold is associated to the inhibitory action of CTLA-4 which depends in turn on TCR and CD28 activation. This induces a stimulation-inhibition cycle which is downstream-propagated throughout the network. Consequently, the threshold *d_CT_*_LA4_ leading to regulation is determined by the expression level attained by activation inducers at a given phase of this cycle.

An analysis of the values of *d_CTLA_*_4_ leading to states of sustained or regulated activation as a function of the stimulation time, τ*_stim_*, is presented in **Figure 5**. We observe that no activation arises for τ*_stim_* < 7 units, while sustained activation ensues for 7 ≤ τ*_stim_* ≤ 10 units, and regulated activation occurs for τ*_stim_* > 10 units. The stage of no activation is associated with insufficient priming time by TCR and CD28. Sustained activation, independent of *d_CTLA_*_4_, is due to the fact that signaling from TCR and CD28 propagates downstream during a certain time without activating CTLA-4. On the other hand, regulated activation is driven by two conditions: first, that τ*_stim_* is long enough, and second, that CTLA-4 activity persists longer than those of activation-inducing elements ( τ*_CTLA_*_4_ = 1/*d_CTLA_*_4_ > 1). In other words, CTLA-4 should be functional long enough to overcome a threshold level and its activity should be maintained to perform inhibition. Therefore, regulated activation persists for even longer values of τ*_stim_* during which *d_CTLA_*_4_ displays a quasi-periodic behavior with slight variations centered at *d_CTLA_*_4_ ~ 0.5. The oscillation of the regulation threshold can be explained by considering that the expression of CTLA-4 depends on activation of the TCR and CD28. Once expressed, CTLA-4 initiates inhibition of signaling, which reduces its own expression and therefore allows activation again; this induces a stimulation-inhibition cycle which is downstream-propagated throughout the network. As a consequence, the threshold value of *d_CTLA_*_4_ leading to regulation will depend on the expression level attained by activation inducers at a given phase of the cycle.

### 3.5 Incomplete Activation Promotes T Cell Anergy With Abnormal Metabolic Profiles

Variable levels of stimulation may originate from the progressive engagement of TCR to MHC-antigen complexes, the lack of a minimal half-life of interaction, diverse levels of antigen concentration, mutations in both cell receptors, etc. (63, 72, 73). Incomplete stimulation leads T cells to anergic states (14, 15, 74) and has been associated with mechanisms of tolerance “(adaptive tolerance”) or antigen presentation failure. On the other hand, defective CD28 co-stimulation allows the expression of the N-Myc Downstream Regulated 1 (NDRG1) protein, leading to anergy (75–77). The effect of different levels of stimulation on the downstream expression of the network components was determined. To simulate these phenomena, we considered initial conditions in which either TCR or CD28 were subjected to a “strong” or a “weak” stimulation. A first case considers *A_MHC_*_/_ *_A_* = 1 and *A_CD_*_8086_ =0.5, and a second one, A*_MHC_*_/_ *_A_* = 0.5 and A*_CD_*_8086_ = 1.

**Figure 6:**
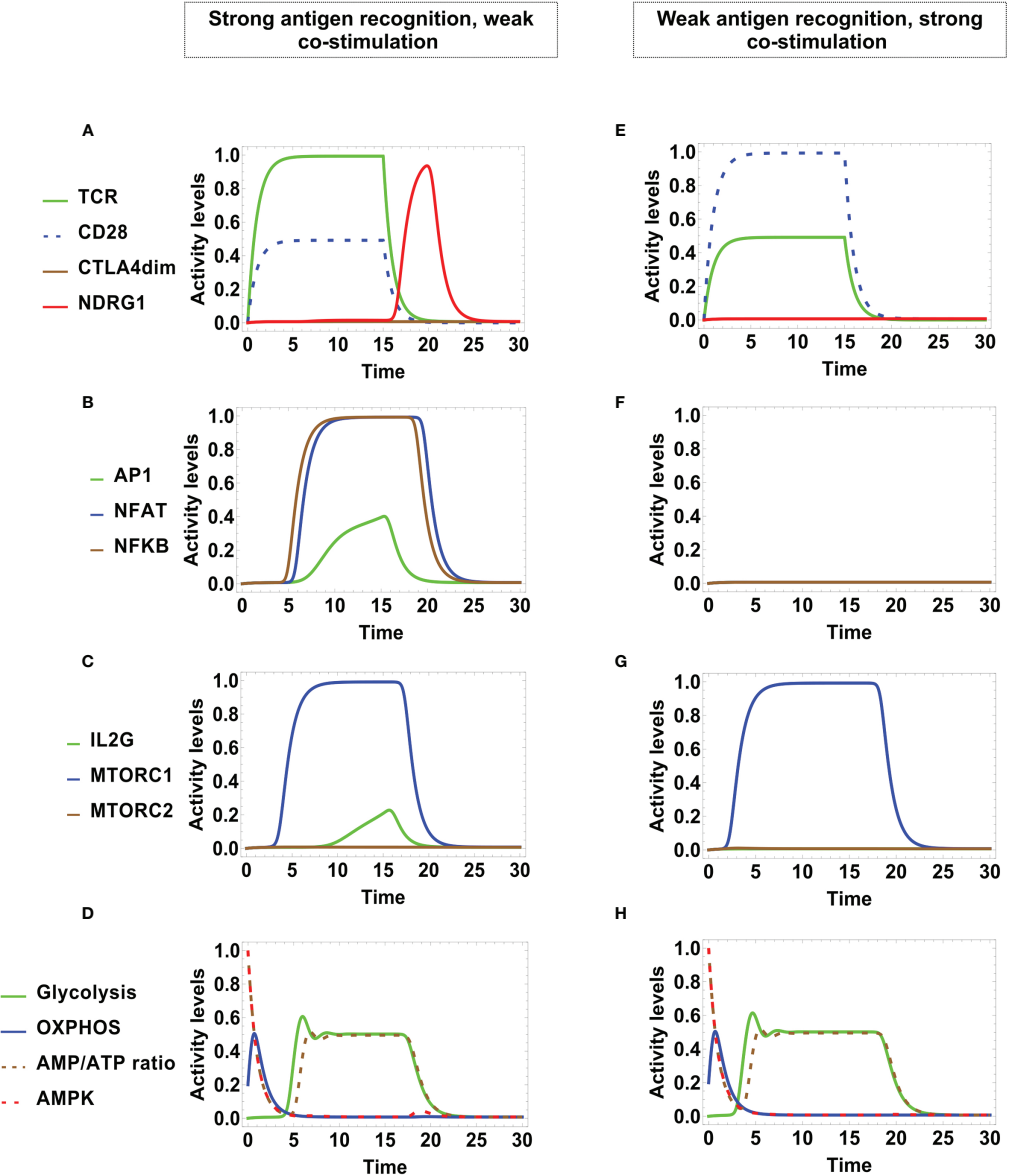
Dynamics of transcription factors and metabolism after incomplete stimulation during an activation time τ*_MHC_*_/_ *_A_* = τ*_CD_*_8086_ = 15 units. Left panel: Anergy is induced by strong TCR stimulation and weak CD28 co-stimulation (A*_MHC_*_/_ *_A_* = 1 and A*_CD_*_8086_ = 0.5). **(A)** TCR and CD28 are transiently activated, decaying both at time τ*_MHC_*_/_ *_A_* = τ*_CD_*_8086_. At this time the anergy factor NDRG1 is temporarily expressed. **(B)** NFAT, NFκB, and AP-1 are down-regulated by the inhibitory action of NDRG1. **(C)** Similarly, mTORC1 and IL2 are only transiently expressed. **(D)** The metabolic profile is similar to that associated to regulated activation induced by CTLA-4 (**Figure 4H**). Right panel: Anergy is induced by weak TCR stimulation and strong CD28 co-stimulation (A*_MHC_*_/_ *_A_* = 0.5 and A*_CD_*_8086_ = 1. **(E)** TCR and CD28 show full and low -level activation, respectively, both decaying at time τ*_MHC_*_/_ *_A_* = τ*_CD_*_8086_; however, the anergy factor NDRG1 remains unexpressed. **(F)**. NFAT, NFκB, and AP-1 are unexpressed. **(G)** mTORC1 is fully activated, but decays along the antigenic stimulation. **(H)** The metabolic profile is similar to that associated to regulated activation induced by CTLA-4 (**Figure 4H**).

The left-hand side of **Figure 6** shows the activation dynamics arising from a strong signaling from TCR and a weak co-stimulation through CD28. TCR and CD28 show full and partial activation, respectively. Low levels of AP-1 and interleukin 2 are transiently expressed. NDRG1 is expressed at a later time, coinciding with the activity decay of TCR and CD28 **(** **Figure 6A** **)**. As before, this conduces to a full but transient expression of NFAT and NFκB, and a very small level of AP-1 (**Figure 6B**). A similar behavior if shown by mTORC1, and IL-2 is strongly suppressed (**Figure 6C**). Notably, the metabolic pattern is identical to that obtained in the case of regulated activation although in this case activity of CTLA-4 is not induced (**Figure 4E** and **Figure 6D**). The former results are consistent with reports indicating that TCR binding in the absence of CD28 ligation results in either apoptosis or a state of anergy that does not involve CTLA-4. Such anergic T cells are unable to produce IL-2 or proliferate on subsequent stimulation, even in the presence of co-stimulation (74, 78).

In the right-hand side of **Figure 6** we show the alternative dynamics arising from a weak signaling of TCR, but a strong co-stimulation by CD28. In this case, TCR and CD28 are transiently expressed at respective low and high levels, but they subsequently decay independently of the action of CTLA-4 or the anergy factor NDRG1 (**Figure 6E**). On the other hand, no transcription factors are induced (**Figure 6F**) neither IL-2, although mTORC1 is transiently expressed (**Figure 6G**). Here, the metabolism profile (**Figure 6H**) also coincides with that obtained for regulated activation **(** **Figure 4H** **)**.

### 3.6 Effector T CD4 Cell Phenotypes Display Specific Metabolism Requirements

It is widely documented that the different effector phenotypes such as Th1, Th2, Th17, and Treg present different energy requirements (56, 79, 80), and studies aimed to elucidate the intricate links between lymphocyte activation and metabolic reprogramming are needed (27). We simulated the individual conditions required for the differentiation of naive T cells into Th1, Th2, Th17 and Treg phenotypes, determined by the presence of the appropriate external cytokines.

Differentiation toward the effector Th cell lineages Th1, Th2, and Th17 is known to be reliant on mTOR activity, while inhibition of mTOR with rapamycin has been shown to favor Treg cell differentiation (56, 80, 81). mTOR complex1 (mTORC1) is formed with the scaffolding protein regulatory associated protein of mTOR (RAPTOR), while mTOR complex 2 (mTORC2) uses Rapamycin-insensitive companion of mammalian target of rapamycin (RICTOR) as a scaffold. All effector lineages, including Th2 cells, require mTORC1 activation (82, 83). Treg cells are a particular case in which the cell uses glycolysis to grow size and replicate; however, at stable stages they predominantly express Foxp3, which is a direct activator of AMPK and OXPHOS activity. This establishes metabolism as a key factor for the correct function of Treg cells (56).

**Figure 7:**
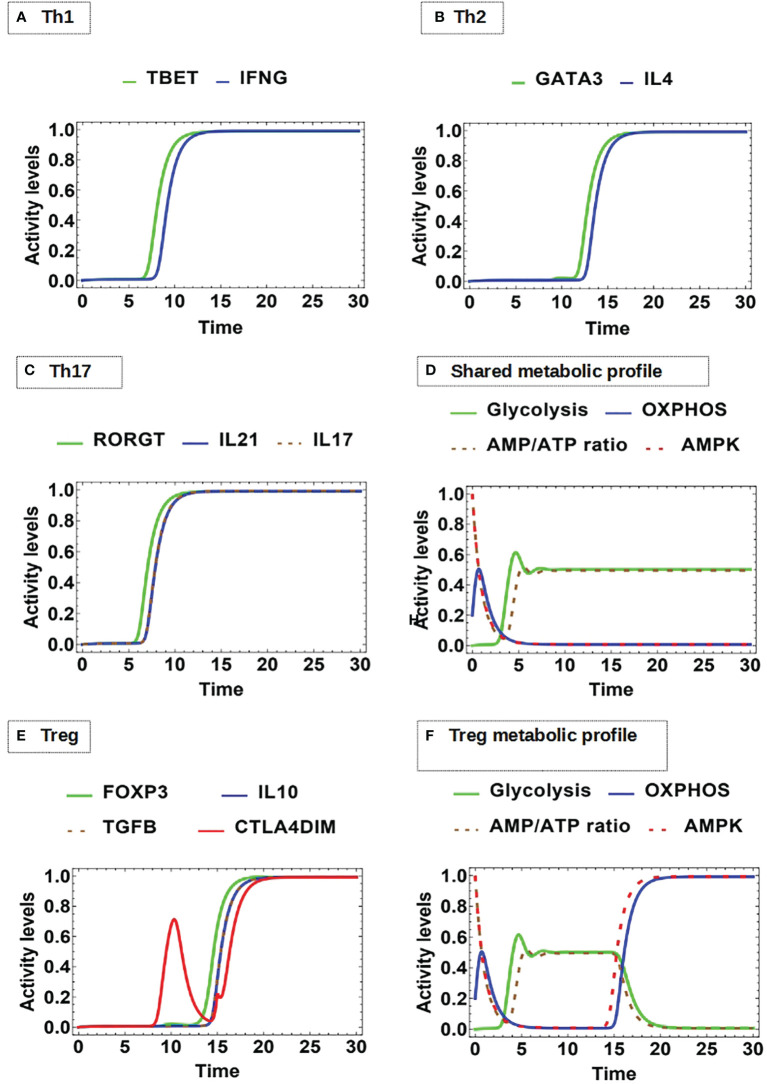
Dynamics of master transcription factors and interleukin production by effector cells under optimal antigenic recognition and low-level activity of CTLA-4: **(A)** Th1 profile: Sustained expression of T-bet and INF-gamma production, **(B)** Th2 profile: Sustained expression of GATA3 and IL-4 production, **(C)** Th17 profile: Sustained expression of RORγT, as well as Il-17 and IL-21 production. **(D)** The Th1, Th2, and Th17 phenotypes display an initial transient OXPHOS phase and then a stable glycolytic metabolism. **(E)** Treg profile: Sustained expression of Foxp3 and dimeric CTLA-4 with joint production of IL-10 and TGF-β. **(F)** Treg displays an initial transient OXPHOS phase followed by glycolytic metabolism; however, with the course of time this is replaced by a metabolism based on OXPHOS.

Modeling of differentiation shows that, after activation, production of the characteristic cytokines starts first for Th1 and Th17 (**Figures 7A, C**), while the Th2 cytokines are induced at later times (**Figure 7B**). As shown before, OXPHOS is transiently upregulated upon activation (**Figures 4D** and **7D**). Next, the three cell phenotypes develops a predominant and stable glycolytic profile. Instead, Treg differentiation shows the production of IL-10 and TGFβ at later times compared to Th1 and Th17 cytokines (**Figure 7E**). Interestingly, the simulation shows that glycolytic activity is followed by a strong and sustained polarization to OXPHOS, corresponding with AMPK activity (**Figure 7F**). This metabolic behavior could allow Treg cells to increase the pool of amino acids required for the synthesis of structural proteins, cell growth for cell clonal expansion, and the production of diverse metabolites necessary to carry out function. However, as the expression of Foxp3 stimulates AMPK, it induces again a polarization to OXPHOS leading cells to a stable regulatory stage. On the other hand, the model is in agreement with the observation that the functional form of CTLA-4 (CTLA-4dim) is upregulated and is constitutively expressed on Treg cells (84) (**Figure 7E**). Thus, the model effectively integrates the pathways that lead to the adjustment of the metabolic profile of different effector phenotypes.

## 4 Discussion

Understanding the function of T CD4 lymphocytes requires dynamic models able to integrate the diversity and connectivity of external and internal signals, the role of their variable levels of expression/function, and the system regulatory mechanisms. Currently, abundant experimental information allows the construction of models taking into account a basic, hierarchic organization of components by identification of those governing the main functional outcomes. Here, we put forward a regulatory network with continuous interactive rules, including a core module encompassing the downstream signals after TCR and CD28 activation along with the antagonist action of CTLA-4, a module including the main components participating in the control of T-cell metabolism, and four differentiation modules associated with the effect of exogenous cytokines leading to effector phenotypes (**Figure 3**). The model reproduces the time course of the main early events of T cell activation, anergy due to the lack of co-stimulation, CTLA-4 checkpoint blockade, cell differentiation, and intrinsic metabolic changes.

The continuous model describes the OXPHOS-glycolysis dynamic adjustment as T cell effector functions develop. Remarkably, the model predicts a transient phase of increased OXPHOS at the onset of activation, followed by its subsequent decrease along with the induction of a glycolytic phase (**Figure 4**). This profile was obtained during differentiation to the Th1, Th2, and Th17 phenotypes (**Figure 7**). These observations are in agreement with experimental data suggesting that OXPHOS produces an ATP reservoir before glycolysis (a much less efficient, but quicker process of ATP production) rises up the levels of metabolites needed for protein synthesis, cell function and growth (53, 70). A possible explanation for this phenomenon is the need for cells to generate a reserve of energy before committing to glycolysis, which implies anabolism in order to produce metabolites for the support of proliferation and function (53). Our model also proposes that in order for the cells to stabilize glycolytic activity, it is necessary to reach an energy balance between the feeding of the metabolic pathways positively regulated by glycolysis, and the AMP/ATP ratio available in the intracellular medium. On the other hand, the model does not predict differences in the metabolism kinetics of Th1, Th2 and Th17 phenotypes. This effect may depend on additional microenvironmental conditions that may be included in the network. In contrast, the glycolysis phase is subsequently reduced during differentiation of Treg cells, as their metabolism is eventually polarized towards OXPHOS (**Figure 7**). The master transcription factor of the Treg lineage (Foxp3) represses glycolytic gene transcription through the upregulation of CTLA-4 and AMPK (56, 85–87). It is suggested that OXPHOS favors the suppressor function of Treg cells, although they may reenter glycolysis when they initiate clonal expansion or migratory activities (51, 56). The continuous model might simulate oscillations of metabolism in changing microenvironmental conditions (work in progress).

The incorporation of two nodes representing the MHC-antigen complex and CD80/86 as inputs of the system allows the assignment of variable levels of functional stimulation and co-stimulation (**Figure 3**). Inhibition of the anergy factor NDRG1 by CD28 as well as IL-2 downstream signaling is required for lymphocyte activation (76) and thus, weak co-stimulation drives the cell into anergy (14, 15, 74, 88). Modeling shows that, when TCR is strongly stimulated and co-stimulation through CD28 is weak, the anergy factor NDRG1 is expressed and anergy emerges. In the anergic state glycolysis is inhibited. In the opposite case, when the TCR signal is weak and CD28 is strongly activated, cells reach a final state in which AP-1, NFAT and NFκB are not produced and the IL-2 gene is not activated. However, activity of mTORC1 is induced along with a transient glycolysis state (**Figure 6**). A study by Zheng et al. has shown that cells induced to anergy display transient increases of expression of the amino acid transporter CD98 and the transferrin receptor CD71, both membrane molecules associated to glycolysis (89). Experimentally, it would be expected to observe a transient activation of the AKT-mTORC1 circuit, along with the activity of enzymes pertaining to the glycolytic pathway in anergic cells. Thus, the model is congruent with the induction of anergy and abnormal metabolic profiles as a result of incomplete stimulation.

The inhibitory action of CTLA-4 is induced after lymphocyte activation and implicitly leads to anergy by competition with CD28 for binding to CD80/86. CTLA-4 has much higher affinity for CD80/86 than CD28. In this panorama, CTLA-4 increases its expression in the cell membrane when the immunological synapse has been maintained during sufficient time, raising the threshold for T-cell responses (10, 90–93). In our model, CTLA-4 is inherently induced by activation signaling; however, the model predicts an initial window over which only sustained activation is observed, because short activation times may be sufficient to activate the T cell but not CTLA-4. The window would allow the initial full activation of the network before turning-on regulatory mechanisms. Thereafter, the inhibitory effect of CTLA-4 depends on the combination of its characteristic time of activity (defined by τ*_CTLA_*_4_ = 1/*d_CTLA_*_4_) and the extent of TCR/CD28 stimulation (**Figure 5**). Thus, a high decay rate allows a sustained T-cell activation. Instead, when the CTLA-4 decay rate is lower (and thus its function is allowed) checkpoint blockade ensues (**Figure 4**). Interestingly, the model predicts that the value of the threshold for the inhibitory action of CTLA-4 oscillates as a function of the TCR and CD28 activation time (**Figure 5**). A relationship between the intensity of antigen stimulation and the activity of CTLA-4 has been suggested by studies showing that the level of translocation of CTLA-4 to the immunological synapse was sensitive to variations in the strength of the TCR signal, and suggesting that CTLA-4 preferentially inhibits the T cell response under conditions of potent TCR-peptide/MHC interactions. Results of modeling agrees with this hypothesis. Diminishing the advantage of highly responding cells, CTLA-4 would avoid that these cells might quickly out-compete clones with weaker responses, allowing for greater representation of cells bearing medium/low affinity TCR’s. In this view, CTLA-4 would favor a greater diversity of the T cell response to a complex set of antigens, by acting at early stages of the cell activation. This effect could be important in the elaboration of a protective T cell response (94).

As expected, activation of CTLA-4 contributes to negatively regulate glycolysis, bringing general metabolism to baseline levels. This is consistent with experimental studies reporting that CTLA-4 blocks the positive regulation of glycolysis through the inhibition of CD28 and AKT pathways (95–97). The glycolysis decline of anergic cells is similar, but not identical, to that displayed during the CTLA-4 checkpoint blockade. The checkpoint blockade is induced by the inhibition of signaling at several levels in the network and is the quickest pathway of glycolysis inhibition (**Figure 4**). In the case of anergy induced by weak antigen stimulation or co-stimulation, glycolysis decay is due to the lack of activity of the IL-2/CD25 axis (**Figures 6C, G**) induced by incomplete stimulation and the activity of NDRG1, respectively (**Figure 3**). In agreement with Frauwirth and cols., CTLA-4 is not activated in these conditions (78) (**Figures 6A, E**).

The continuous model proposed here may constitute a relevant step for the comprehensive integration of experimental information on the mechanism behind activation and function of T CD4 cells. Additional factors with a role in the response to diverse stimuli and microenvironmental conditions may be incorporated, including different levels of ligand interactions and times of activity of key players. It is also useful for the understanding of the emerging metabolic requirements of different functional stages of T CD4 cells.

## Data Availability Statement

The original contributions presented in the study are included in the article/**Supplementary Material**. Further inquiries can be directed to the corresponding authors.

## Author Contributions

DM-M, CV, LM, and LH contributed to the conception of the model. DM-M and LH designed the regulatory network. DM-M and CV constructed the logical propositions, conducted numerical experiments, and performed the analysis of the system dynamics. All authors contributed to the interpretation of results. All authors participated in manuscript writing.

## Funding

This work was supported by Programa de Apoyo a Proyectos de Investigación e Innovación Tecnológica of the Universidad Nacional Autónoma de México (grant number IN215820 to LH, and IN202721 to LM) and a postdoctoral fellowship from CONACYT (CVU number 555239 to DM-M).

## Conflict of Interest

The authors declare that the research was conducted in the absence of any commercial or financial relationships that could be construed as a potential conflict of interest.

## Publisher’s Note

All claims expressed in this article are solely those of the authors and do not necessarily represent those of their affiliated organizations, or those of the publisher, the editors and the reviewers. Any product that may be evaluated in this article, or claim that may be made by its manufacturer, is not guaranteed or endorsed by the publisher.
