# Supplementary material for: Continuous Modeling of T CD4 Lymphocyte Activation and Function
Source: Front Immunol. 2021 Nov 5;12:743559. doi: 10.3389/fimmu.2021.743559 (PMC8602102; doi:10.3389/fimmu.2021.743559)
Supplement: Supplementary file 1 [file DataSheet_1.pdf]

#Boolean rules for the T CD4 cell activation network#

Targets, factors

#General Core Inputs#

CD8086, CD8086

TCR, TCR & ! CTLA4dim
CD28, CD8086 & ! CTLA4dim
AP1, rasGTPr
CD25, IL2G & ! CTLA4dim
IL2G, NFAT & AP1 & ! NDRG1 & ! RORGT & ! IL21e & ! IL21| NFKB & NFAT & AP1 & ! NDRG1 & ! RORGT & ! IL21e & ! IL21 | STAT5 & NFAT & AP1 & ! NDRG1 & ! RORGT & ! IL21e & ! IL21
ZAP70, (TCR & LCK) & ! CTLA4dim
STAT5, CD25 & ! CTLA4dim
NFAT, CA | CA & AKT
NFKB, (PKC) | (PKC & PDK1)
AKT, (CD28 & ! CTLA4dim) | PDK1
CTLA4, (IL2G & ZAP70)
CTLA4dim, (CTLA4 & ! CD8086) | (FOXP3 & TGFB)
BCL2, AKT
NDRG1, NFAT & ! AKT
DAG, (PLC) | (AKT & PLC)
SOS, CD28 | CD28 & CD25
rasGTPr, LAT & SOS & DAG
LCK, TCR & ! CTLA4dim
PDK1, CD25 | CD28
LAT, ZAP70
PLC, ZAP70
PIP2, PLC
PIP3, PIP2
CA, PIP3
PKC, DAG

#Metabolism#

MTOR, CD25 | AKT
MTORC1, MTOR & ! AMPK
MTORC2, MTOR & AMPK | MTOR & IL4e
LKB1, AKT & AMPATPratio
AMPK, (LKB1 & ! MTORC1) | (CA & AMPATPratio & ! MTORC1) | (AKT & AMPATPratio & ! MTORC1) | FOXP3
Glycolysis, MTORC1 & ! AMPATPratio
OXPHOS, AMPK
AMPATPratio, Glycolysis & ! OXPHOS

#Phenotype inputs#

```
IFNGe, IFNGe
IL12e, IL12e
#Phenotype nodes#
TBET, (MTORC1 & IL12e & IFNGe & NFKB & NFAT & AP1) & !IL4 & !IL10 & !GATA3
IFNG, (TBET & AP1 & NFAT)

#Phenotype inputs#

IL4e, IL4e
#Phenotype nodes#
GATA3, ((MTORC2 & IL4e & STAT5 & NFAT) & (!TBET & !TGFB & !IFNG)
IL4, (GATA3 & !TBET) & !IFNG

#Phenotype inputs#

TGFBe, TGFBe
IL10e, IL10e
#Phenotype nodes#
FOXP3, ((NFAT & STAT5 & AP1 & IL2G & TGFBe & IL10e) | (IL10 & TGFBe & IL10e & CTLA4)
| (TGFB & TGFBe)) & ! IFNG
IL10, FOXP3 & TGFBe
TGFB, FOXP3

#Phenotype inputs#

IL21e, IL21e
TGFBe,TGFBe
#Phenotype nodes#
RORGT, (CD28 & MTORC1 & IL21e & TGFBe) & ! (TBET | FOXP3 | GATA3)
IL21, ( IL21e | RORGT ) & ! (IFNG | IL4 | IL10 )
IL17, RORGT
```
