# Supplementary material for: Continuous Modeling of T CD4 Lymphocyte Activation and Function
Source: Front Immunol. 2021 Nov 5;12:743559. doi: 10.3389/fimmu.2021.743559 (PMC8602102; doi:10.3389/fimmu.2021.743559)
Supplement: Supplementary file 2 [file DataSheet_2.pdf]

```
(*ODEs list for the network components*)
```

```
(*Traditional form*)
```

$$\frac{\partial \mathrm{TCR}}{\partial t} = \frac{1}{\exp(-b(\mathrm{WTCR}(t) - .5)) + 1} - \mathrm{DTCR}\ \mathrm{TCR}(t)$$

$$\frac{\partial \mathrm{CD28}}{\partial t} = \frac{1}{\exp(-b(\mathrm{WCD28}(t) - .5)) + 1} - \mathrm{DCD28}\ \mathrm{CD28}(t)$$

$$\frac{\partial \mathrm{CD8086E}}{\partial t} = \frac{1}{\exp(-b(\mathrm{WCD8086E}(t) - .5)) + 1} - \mathrm{DCD8086E}\ \mathrm{CD8086E}(t)$$

$$\frac{\partial \mathrm{AP1}}{\partial t} = \frac{1}{\exp(-b(\mathrm{WAP1}(t) - .5)) + 1} - \mathrm{DAP1}\ \mathrm{AP1}(t)$$

$$\frac{\partial \mathrm{CD25}}{\partial t} = \frac{1}{\exp(-b(\mathrm{WCD25}(t) - .5)) + 1} - \mathrm{DCD25}\ \mathrm{CD25}(t)$$

$$\frac{\partial \mathrm{IL2G}}{\partial t} = \frac{1}{\exp(-b(\mathrm{WIL2G}(t) - .5)) + 1} - \mathrm{DIL2G}\ \mathrm{IL2G}(t)$$

$$\frac{\partial \mathrm{MTOR}}{\partial t} = \frac{1}{\exp(-b(\mathrm{WMTOR}(t) - .5)) + 1} - \mathrm{DMTOR}\ \mathrm{MTOR}(t)$$

$$\frac{\partial \mathrm{ZAP70}}{\partial t} = \frac{1}{\exp(-b(\mathrm{WZAP70}(t) - .5)) + 1} - \mathrm{DZAP70}\ \mathrm{ZAP70}(t)$$

$$\frac{\partial \mathrm{STAT5}}{\partial t} = \frac{1}{\exp(-b(\mathrm{WSTAT5}(t) - .5)) + 1} - \mathrm{DSTAT5}\ \mathrm{STAT5}(t)$$

$$\frac{\partial \mathrm{NFAT}}{\partial t} = \frac{1}{\exp(-b(\mathrm{WNFAT}(t) - .5)) + 1} - \mathrm{DNFAT}\ \mathrm{NFAT}(t)$$

$$\frac{\partial \mathrm{NFKB}}{\partial t} = \frac{1}{\exp(-b(\mathrm{WNFKB}(t) - .5)) + 1} - \mathrm{DNFKB}\ \mathrm{NFKB}(t)$$

$$\frac{\partial \mathrm{AKT}}{\partial t} = \frac{1}{\exp(-b(\mathrm{WAKT}(t) - .5)) + 1} - \mathrm{DAKT}\ \mathrm{AKT}(t)$$

$$\frac{\partial \mathbf{CTLA4}}{\partial t} = \frac{1}{\exp(-b(\mathrm{WCTLA4}(t) - .5)) + 1} - \mathrm{DCTLA4}\ \mathrm{CTLA4}(t)$$

$$\frac{\partial \mathbf{CTLA4DIM}}{\partial t} = \frac{1}{\exp(-b(\mathrm{WCTLA4DIM}(t) - .5)) + 1} - \mathrm{DCTLA4DIM}\ \mathrm{CTLA4DIM}(t)$$

$$\frac{\partial \mathbf{BCL2}}{\partial t} = \frac{1}{\exp(-b(\mathrm{WBCL2}(t) - .5)) + 1} - \mathrm{DBCL2}\ \mathrm{BCL2}(t)$$

$$\frac{\partial \mathbf{NDRG1}}{\partial t} = \frac{1}{\exp(-b(\mathrm{WNDRG1}(t) - .5)) + 1} - \mathrm{DNDRG1}\ \mathrm{NDRG1}(t)$$

$$\frac{\partial \mathbf{DAG}}{\partial t} = \frac{1}{\exp(-b(\mathrm{WDAG}(t) - .5)) + 1} - \mathrm{DDAG}\ \mathrm{DAG}(t)$$

$$\frac{\partial \mathbf{SOS}}{\partial t} = \frac{1}{\exp(-b(\mathrm{WSOS}(t) - .5)) + 1} - \mathrm{DSOS}\ \mathrm{SOS}(t)$$

$$\frac{\partial \mathbf{RASGTPR}}{\partial t} = \frac{1}{\exp(-b(\mathrm{WRASGTPR}(t) - .5)) + 1} - \mathrm{DRASGTPR}\ \mathrm{RASGTPR}(t)$$

$$\frac{\partial \mathbf{LCK}}{\partial t} = \frac{1}{\exp(-b(\mathrm{WLCK}(t) - .5)) + 1} - \mathrm{DLCK}\ \mathrm{LCK}(t)$$

$$\frac{\partial \mathbf{PDK1}}{\partial t} = \frac{1}{\exp(-b(\mathrm{WPDK1}(t) - .5)) + 1} - \mathrm{DPDK1}\ \mathrm{PDK1}(t)$$

$$\frac{\partial \mathbf{MTORC1}}{\partial t} = \frac{1}{\exp(-b(\mathrm{WMTORC1}(t) - .5)) + 1} - \mathrm{DMTORC1}\ \mathrm{MTORC1}(t)$$

$$\frac{\partial \mathbf{MTORC2}}{\partial t} = \frac{1}{\exp(-b(\mathrm{WMTORC2}(t) - .5)) + 1} - \mathrm{DMTORC2}\ \mathrm{MTORC2}(t)$$

$$\frac{\partial \mathbf{LAT}}{\partial t} = \frac{1}{\exp(-b(\mathrm{WLAT}(t) - .5)) + 1} - \mathrm{DLAT}\ \mathrm{LAT}(t)$$

$$\frac{\partial \mathbf{PLC}}{\partial t} = \frac{1}{\exp(-b(\mathrm{WPLC}(t) - .5)) + 1} - \mathrm{DPLC}\ \mathrm{PLC}(t)$$

$$\frac{\partial \mathbf{PIP2}}{\partial t} = \frac{1}{\exp(-b(\mathrm{WPIP2}(t) - .5)) + 1} - \mathrm{DPIP2}\ \mathrm{PIP2}(t)$$

$$\frac{\partial \mathrm{PIP3}}{\partial t} = \frac{1}{\exp(-b(\mathrm{WPIP3}(t) - .5)) + 1} - \mathrm{DPIP3}\ \mathrm{PIP3}(t)$$

$$\frac{\partial \mathrm{CA}}{\partial t} = \frac{1}{\exp(-b(\mathrm{WCA}(t) - .5)) + 1} - \mathrm{DCA}\ \mathrm{CA}(t)$$

$$\frac{\partial \mathrm{PKC}}{\partial t} = \frac{1}{\exp(-b(\mathrm{WPKC}(t) - .5)) + 1} - \mathrm{DPKC}\ \mathrm{PKC}(t)$$

$$\frac{\partial \mathrm{TBET}}{\partial t} = \frac{1}{\exp(-b(\mathrm{WTBET}(t) - .5)) + 1} - \mathrm{DTBET}\ \mathrm{TBET}(t)$$

$$\frac{\partial \mathrm{IFNG}}{\partial t} = \frac{1}{\exp(-b(\mathrm{WIFNG}(t) - .5)) + 1} - \mathrm{DIFNG}\ \mathrm{IFNG}(t)$$

$$\frac{\partial \mathrm{GATA3}}{\partial t} = \frac{1}{\exp(-b(\mathrm{WGATA3}(t) - .5)) + 1} - \mathrm{DGATA3}\ \mathrm{GATA3}(t)$$

$$\frac{\partial \mathrm{IL4}}{\partial t} = \frac{1}{\exp(-b(\mathrm{WIL4}(t) - .5)) + 1} - \mathrm{DIL4}\ \mathrm{IL4}(t)$$

$$\frac{\partial \mathrm{FOXP3}}{\partial t} = \frac{1}{\exp(-b(\mathrm{WFOXP3}(t) - .5)) + 1} - \mathrm{DFOXP3}\ \mathrm{FOXP3}(t)$$

$$\frac{\partial \mathrm{IL10}}{\partial t} = \frac{1}{\exp(-b(\mathrm{WIL10}(t) - .5)) + 1} - \mathrm{DIL10}\ \mathrm{IL10}(t)$$

$$\frac{\partial \mathrm{TGFB}}{\partial t} = \frac{1}{\exp(-b(\mathrm{WTGFB}(t) - .5)) + 1} - \mathrm{DTGFB}\ \mathrm{TGFB}(t)$$

$$\frac{\partial \mathrm{RORGT}}{\partial t} = \frac{1}{\exp(-b(\mathrm{WRORGT}(t) - .5)) + 1} - \mathrm{DRORGT}\ \mathrm{RORGT}(t)$$

$$\frac{\partial \mathrm{IL21}}{\partial t} = \frac{1}{\exp(-b(\mathrm{WIL21}(t) - .5)) + 1} - \mathrm{DIL21}\ \mathrm{IL21}(t)$$

$$\frac{\partial \mathrm{IL17}}{\partial t} = \frac{1}{\exp(-b(\mathrm{WIL17}(t) - .5)) + 1} - \mathrm{DIL17}\ \mathrm{IL17}(t)$$

$$\frac{\partial \mathrm{LKB1}}{\partial t} = \frac{1}{\exp(-b(\mathrm{WLKB1}(t) - .5)) + 1} - \mathrm{DLKB1}\ \mathrm{LKB1}(t)$$

$$\frac{\partial \mathrm{AMPK}}{\partial t} = \frac{1}{\exp(-b(\mathrm{WAMPK}(t) - .5)) + 1} - \mathrm{DAMPK}\ \mathrm{AMPK}(t)$$

$$\frac{\partial \mathrm{Glycolysis}}{\partial t} = \frac{1}{\exp(-b(\mathrm{WGlycolysis}(t) - .5)) + 1} - \mathrm{DGlycolysis}\ \mathrm{Glycolysis}(t)$$

$$\frac{\partial \mathrm{OXPHOS}}{\partial t} = \frac{1}{\exp(-b(\mathrm{WOXPHOS}(t) - .5)) + 1} - \mathrm{DOXPHOS}\ \mathrm{OXPHOS}(t)$$

$$\frac{\partial \mathrm{AMPATPratio}}{\partial t} = \frac{1}{\exp(-b(\mathrm{WAMPATPratio}(t) - .5)) + 1} - \mathrm{DAMPATPratio}\ \mathrm{AMPATPratio}(t)$$
