# Supplementary material for: Continuous Modeling of T CD4 Lymphocyte Activation and Function
Source: Front Immunol. 2021 Nov 5;12:743559. doi: 10.3389/fimmu.2021.743559 (PMC8602102; doi:10.3389/fimmu.2021.743559)
Supplement: Supplementary file 3 [file DataSheet_3.pdf]

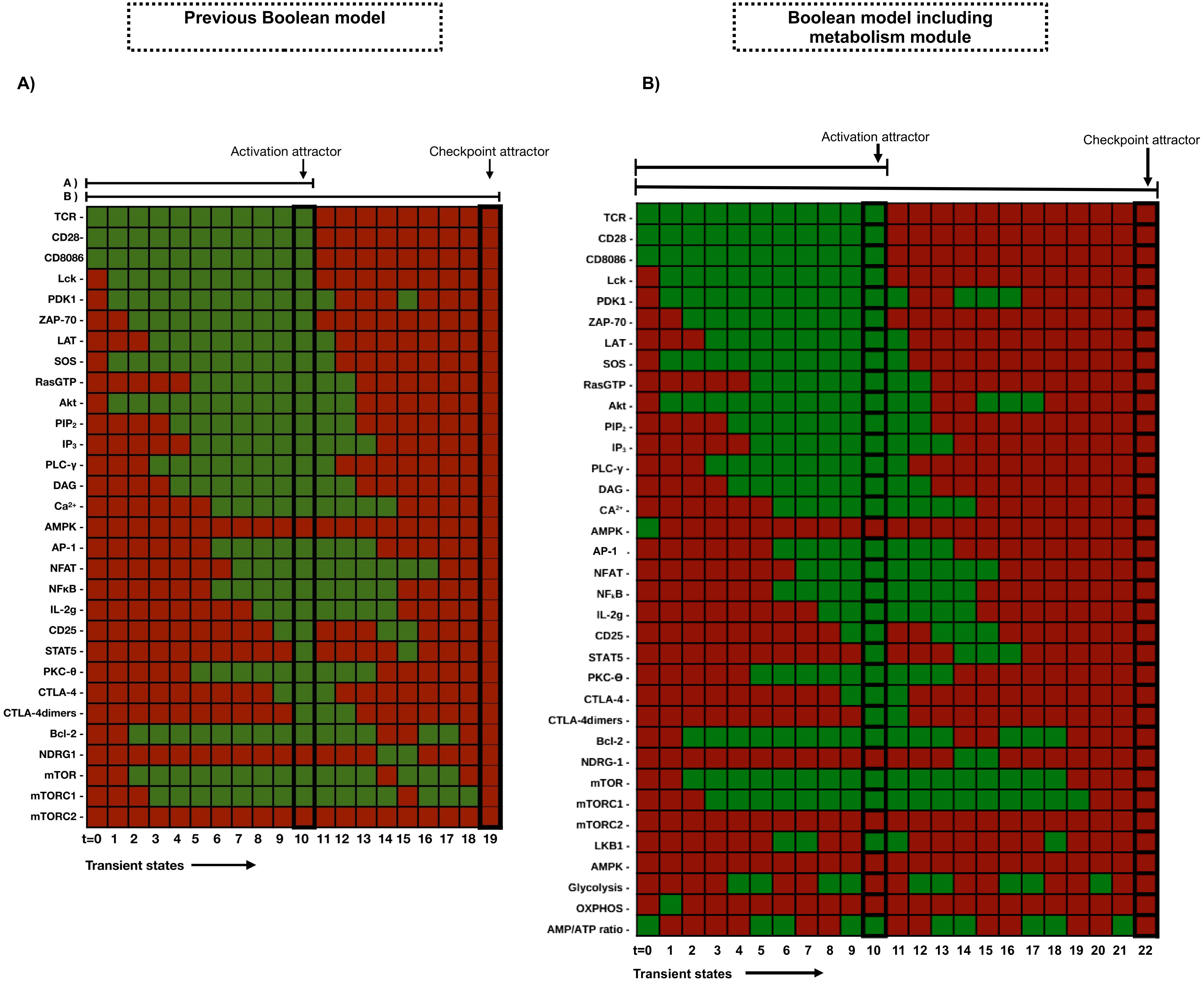

Supplemental material 3. Comparison of the node expression dynamics obtained using a previously published Boolean model (Martínez-Méndez et al., 2020) (A), and the version including the metabolism module, as shown in figures 2 and 3 (B). Both simulations were performed using the same initial conditions. The metabolic module does not change the general behavior of the system; both models produce the same transitory states of activation and final checkpoint attractors.
